# Screening Performance of Anthropometric Indices and Determination of Optimal Cut-Off Values for Identifying Low Muscle Strength in Hospitalized Geriatric Patients

**DOI:** 10.3390/jcm15145420

**Published:** 2026-07-10

**Authors:** Justyna Nowak, Marzena Jabczyk, Michał Skrzypek, Michał Górski, Bartosz Hudzik, Barbara Zubelewicz-Szkodzińska

**Affiliations:** 1 Department of Cardiovascular Disease Prevention, Department of Metabolic Disease Prevention, Faculty of Public Health in Bytom, Medical University of Silesia, 41-902 Bytom, Poland; bhudzik@sum.edu.pl; 2 Department of Nutrition-Related Disease Prevention, Department of Metabolic Disease Prevention, Faculty of Public Health in Bytom, Medical University of Silesia, 41-902 Bytom, Poland; marzena.jabczyk@sum.edu.pl (M.J.); bzubelewicz-szkodzinska@sum.edu.pl (B.Z.-S.); 3 Department of Biostatistics, Faculty of Public Health in Bytom, Medical University of Silesia, 41-902 Bytom, Poland; mskrzypek@sum.edu.pl; 4 Department of Chronic Diseases and Civilization-Related Hazards, Faculty of Public Health in Bytom, Medical University of Silesia in Katowice, 41-902 Bytom, Poland; michal.gorski@sum.edu.pl; 5 Third Department of Cardiology, Silesian Center for Heart Disease, Faculty of Medical Sciences in Zabrze, Medical University of Silesia, 41-800 Zabrze, Poland; 6 Department of Endocrinology, District Hospital, 41-940 Piekary Slaskie, Poland

**Keywords:** low muscle strength, anthropometric measurements, anthropometric indices, older adults, sarcopenia

## Abstract

**Background:** Sarcopenia, characterized by age-related loss of muscle mass and strength, is a growing public health concern requiring early detection; this exploratory study evaluated a range of anthropometric measurements and indices to identify those most suitable for screening low muscle strength in hospitalized geriatric patients and to determine preliminary cut-off values that may support screening in settings with limited access to specialized equipment or trained personnel. **Materials and Methods:** In this cross-sectional study, 390 hospitalized geriatric-ward patients aged ≥ 60 years were included. The median age was 77.00 years (72.00, 82.00), and 258 participants (66.2%) were women. Anthropometric and body composition measurements were performed, and handgrip strength was assessed. Low muscle strength was defined according to The European Working Group on Sarcopenia in Older People 2 (EWGSOP2) criteria. Logistic regression analyses were used to assess associations between anthropometric measurements and low muscle strength, and receiver operating characteristic (ROC) curve analysis was used to evaluate their screening performance. **Results:** Among 390 participants, 67 (17.2%) had low muscle strength. Low muscle strength was associated with older age, lower body weight, BMI (Body Mass Index), fat-free mass, and smaller arm and calf circumferences. Higher arm and calf circumferences were significantly associated with a lower risk of low muscle strength in both women (OR = 0.913 and 0.884) and men (OR = 0.793 and 0.769; all *p* < 0.05). ROC analysis identified optimal screening cut-offs: arm 26.5 cm and calf 31.5 cm in women, and arm 29.5 cm and calf 33 cm in men, showing moderate screening performance, with an area under the ROC curve (AUC) of 0.63–0.78. **Conclusions:** Calf and arm circumferences are simple, quick, and non-invasive measurements that may be useful for screening low muscle strength in hospitalized geriatric patients, particularly in settings with limited access to specialized equipment. These measurements may help identify individuals who require further assessment for sarcopenia according to current clinical guidelines, with age being an important factor to consider. The proposed cut-off values should be considered hypothesis-generating rather than diagnostic thresholds for sarcopenia and require external validation in independent populations before they can be recommended for routine clinical use.

## 1. Introduction

One of the most characteristic changes associated with ageing is the gradual loss of skeletal muscle mass, which contributes to reduced muscle strength and functional capacity [1,2,3]. The term “sarcopenia” was first introduced by Irwin Rosenberg in 1989 and has since become widely used to describe the age-related decline in skeletal muscle mass and strength, leading to impaired daily functioning and increased risk of functional dependence [2].

Over the years, several definitions of sarcopenia have been proposed. In 2010, the European Working Group on Sarcopenia in Older People (EWGSOP) introduced a clinical definition and diagnostic criteria for age-related sarcopenia [4,5]. According to EWGSOP, sarcopenia was defined as a progressive and generalized loss of skeletal muscle mass and strength, associated with adverse outcomes such as physical disability, reduced quality of life, and mortality. The diagnosis was based on the presence of low muscle mass combined with low muscle strength and/or poor physical performance [5]. In 2016, sarcopenia was included in the International Classification of Diseases (ICD-10) under the code M62.84, which further supported its recognition as a distinct clinical condition [6].

As research progressed, the limitations of the 2010 EWGSOP criteria became more evident, particularly because the assessment of muscle mass as the primary diagnostic criterion is difficult to apply routinely in clinical practice [7,8]. In response, the EWGSOP2 group revised the definition and diagnostic algorithm of sarcopenia, placing low muscle strength at the beginning of the diagnostic pathway [8]. Muscle strength is considered a key indicator because it predicts adverse outcomes, including falls, fractures, physical disability, and mortality, more strongly than muscle mass [8]. According to EWGSOP2, low muscle strength indicates probable sarcopenia, while reduced muscle quantity or quality is required to confirm the diagnosis. Poor physical performance is used to identify severe sarcopenia [8].

Sarcopenia, as a progressive loss of skeletal muscle mass and function, is increasingly recognized as an important geriatric syndrome with serious individual and public health consequences. It is associated with multiple adverse outcomes, including physical disability, depression, reduced quality of life, nursing home admission, and increased mortality, which highlights the need for timely identification and appropriate management [9].

Sarcopenia is a prevalent and clinically important condition in older adults, although its reported frequency varies widely depending on region, age, population characteristics, care setting, diagnostic criteria, and cut-off values used [4,10,11,12,13]. Available studies consistently show that sarcopenia is common across community, nursing home, long-term care, and hospital settings, contributing to functional decline, falls, loss of independence, and increased healthcare utilization [4,10,11,12,13].

Although handgrip strength assessment is simpler and more feasible than direct assessment of muscle mass, routine dynamometry may still be limited in some care settings by the lack of equipment, trained personnel, or systematic screening procedures. In this context, simple anthropometric measurements may support initial case-finding by helping to identify older adults who are more likely to have low muscle strength and who may require further assessment according to EWGSOP2 recommendations.

Among simple anthropometric measurements, calf circumference has been widely investigated as a practical marker related to muscle status and sarcopenia screening [14,15,16]. Evidence also suggests that arm circumference, other limb circumferences, and selected anthropometric measurements are associated with handgrip strength in older adults [17] Thus, the present study does not propose an entirely new screening concept. The specific novelty of this work lies in evaluating the screening performance of these anthropometric measurements and indices and deriving sex-specific cut-off values for identifying EWGSOP2-defined low handgrip strength in a hospitalized geriatric population. The aim of this study was to evaluate the screening performance of a range of anthropometric measurements and indices for identifying low muscle strength in older adults and to determine preliminary cut-off values that may support screening in settings with limited access to specialized equipment or trained personnel.

## 2. Materials and Methods

### 2.1. Study Design and Exploratory Approach

This study had an exploratory design and focused on analyzing available anthropometric measurements and indices to identify those that could be useful for preliminary screening of low muscle strength in older adults in facilities without access to specialized equipment, such as handheld dynamometers or bioelectrical impedance analyzers. Particular attention was given to parameters that can be assessed without specialized devices or time-consuming procedures.

### 2.2. Ethics Approval

The study was conducted in accordance with the ethical principles of the Declaration of Helsinki and was approved by the Bioethics Committee of the Medical University of Silesia (approval number: KNW/0022/KB1/53/14, approval date: 3 June 2014). All participants gave their informed consent before joining the study. They were fully informed about the purpose, scope, and procedures of the study, and were assured that they could withdraw at any time without giving a reason and without affecting their medical care. The study procedures involved minimal risk and did not add any burden beyond standard clinical care. All collected data were anonymized, securely stored, and handled according to data protection laws, with no personal identifiers kept. The research team followed ethical standards throughout the study.

### 2.3. Study Population

**Figure 1:**
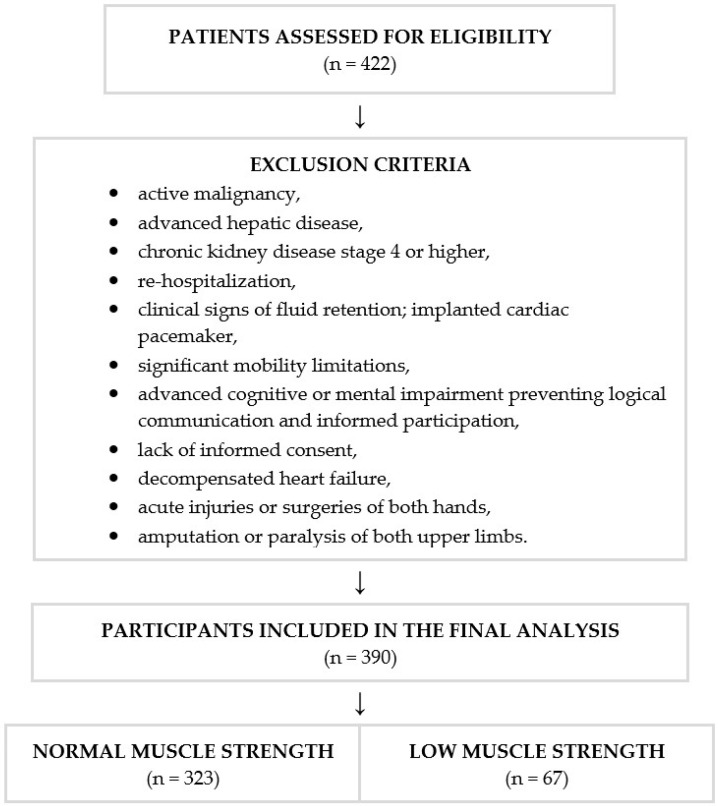
Participant flow diagram.

The study was conducted from September 2014 to December 2016 among patients aged 60 years and older admitted to a geriatric ward in southern Poland for diagnostic and therapeutic management of various medical conditions. Of the 422 patients initially assessed for eligibility, 32 were excluded according to the predefined exclusion criteria described below; therefore, 390 participants were included in the final analysis (Figure 1).

Patients were excluded from the final analysis [18,19] if they met any of the following criteria: active malignancy (under treatment or with a confirmed diagnosis), advanced hepatic disease (e.g., liver cirrhosis), chronic kidney disease stage 4 or higher (eGFR < 30 mL/min/1.73 m^2^), re-hospitalization, clinical signs of fluid retention at the time of assessment (e.g., lower limb edema), presence of an implanted cardiac pacemaker, significant mobility limitations, advanced cognitive or mental impairment preventing logical communication and informed participation in the study, or lack of informed consent. Additionally, due to the known influence of fluid overload on body weight and bioelectrical impedance measurements, individuals with decompensated heart failure were excluded to ensure reliable, edema-free body composition analysis. Due to the requirements of the muscle strength measurement, acute injuries or surgeries of both hands, as well as amputation or paralysis of both upper limbs, were also considered to be exclusion criteria.

Based on hand grip strength, the study population was divided into two groups. The first group, defined as “Normal Muscle Strength,” included 323 patients without low muscle strength. “Low muscle strength”, consisted of 67 patients with low muscle strength. Low muscle strength was defined according to the EWGSOP2 criteria. The EWGSOP2 sarcopenia cut-off points for low muscle strength were applied, with grip strength thresholds of <27 kg for men and <16 kg for women [8].

### 2.4. Sample Size Estimation

The minimum required sample size was calculated assuming an area under the curve (AUC) of 0.70, a significance level of 0.05, and a statistical power of 0.80. A control-to-case ratio of 4.5 was applied. The estimated minimum sample size was 19 cases and 86 controls. Our dataset met these requirements, including after stratification by sex. Calculations were performed using the pROC package [20] in R software, version 4.5.1 (R Foundation for Statistical Computing, Vienna, Austria) [21].

### 2.5. Laboratory Assessments

As part of the clinical evaluation, data were collected on participants’ age, sex, and medical history. Anthropometric and laboratory parameters were also assessed. All blood samples were taken in the morning following an overnight fast. The biochemical analyses included measurements of fasting glucose, total cholesterol, HDL cholesterol, LDL cholesterol, non-HDL cholesterol, triglycerides, total protein and C-reactive protein. Kidney function was evaluated using estimated glomerular filtration rate (eGFR) and serum creatinine levels. Liver function tests included aspartate aminotransferase (AST), alanine aminotransferase (ALT), and gamma-glutamyl transferase (GGT). Additional parameters measured were serum albumin, vitamin D, and thyroid-stimulating hormone (TSH).

### 2.6. Anthropometric Measurements and Body Composition Analysis

Baseline nutritional assessments were performed in the morning while patients were fasting, barefoot, and dressed in light clothing. Standardized procedures and validated instruments were used for all measurements. Body weight was assessed using the Tanita BC 420 S MA scale (Tanita Corporation, Tokyo, Japan) with an accuracy of 0.01 kg. Height was measured with the Tanita HR 100 stadiometer (Tanita Corporation, Tokyo, Japan), accurate to 0.05 cm. Waist, hip, mid-arm, and calf circumferences were measured using the Seca 203 measuring tape (seca GmbH & Co. KG., Hamburg Germany), with a precision of 1 mm.

Body height was measured in a standing position, with participants barefoot, feet placed together, arms relaxed along the body, and the head maintained in a neutral position while looking straight ahead. Waist circumference was measured at the umbilical level, within the area between the lower margin of the rib cage and the iliac crest, with the tape positioned horizontally around the trunk and the measurement taken after a gentle expiration. Hip circumference was assessed below the iliac crests at the level of the greatest posterior protrusion of the buttocks.

Mid-arm circumference was measured on the non-dominant upper limb, with the arm relaxed alongside the trunk, at the midpoint between the acromion process of the scapula and the olecranon process of the ulna. Calf circumference was measured on the left lower limb at the point of maximum calf girth, with participants standing upright, feet slightly apart, and body weight evenly distributed on both legs. Before calf circumference assessment, the lower limbs were inspected for edema.

Each anthropometric measurement was performed once, and the obtained value was used in the analysis. Formal assessor blinding was not applied; however, participants were classified by muscle strength status only after all anthropometric and handgrip strength measurements had been completed.

Body composition was evaluated using the Tanita BC 420 S MA Body Composition Analyzer, a bioelectrical impedance analysis device used to estimate body composition parameters, including body fat percentage, fat mass (kg), muscle mass (kg), fat-free mass (kg), and visceral fat level.

### 2.7. Anthropometric Calculations

The following indices were calculated using established formulas presented in Table 1.

### 2.8. Grip Strength Measurement

To assess hand grip strength, we used the Smedley dynamometer (certified under MDD 93/42 EEC). Handgrip strength was assessed using a standardized protocol. Participants were seated on a chair without armrests, with their feet placed flat on the floor. The upper limbs were positioned neutrally, with the shoulders and forearms relaxed. A calibrated hand dynamometer was used for the assessment, with the grip width set to position 2 for all participants to ensure uniformity.

Each participant performed three consecutive grip strength trials with each hand, alternating sides between attempts. For analysis purposes, the highest grip strength value obtained from all available measurements (across both hands) was used, provided that at least two valid measurements were recorded for one hand [27,28,29].

### 2.9. Statistical Analysis

All statistical analyses were performed using R software, version 4.5.1 (R Foundation for Statistical Computing, Vienna, Austria). The Shapiro–Wilk test was applied to assess the normality of the distribution of continuous variables. Variables following a normal distribution were presented as mean ± standard deviation (SD), while those with a non-normal distribution were reported as medians with lower and upper quartiles.

Group comparisons were conducted using the independent *t*-test for normally distributed data and the Mann–Whitney U test for non-normally distributed data. Categorical variables were compared using the chi-squared test, and Fisher’s exact test was used when expected cell counts were small.

To examine associations between continuous variables, Pearson’s correlation coefficient was used for normally distributed data, and Spearman’s rank correlation coefficient was used otherwise. To account for multiple hypothesis testing, a false discovery rate correction was applied, and adjusted *p* values are reported as q values in Tables 2–4.

Receiver Operating Characteristic (ROC) curve analysis was employed to evaluate the ability of selected anthropometric parameters to identify low muscle strength. The area under the ROC curve (AUC) was calculated to assess the discriminative ability of each anthropometric parameter to identify individuals with low muscle strength, and the Youden index was used to determine the optimal cut-off values. Additionally, sensitivity, specificity, positive predictive value (PPV), and negative predictive value (NPV) were calculated.

To assess the strength of the association between individual anthropometric measurements or indices and low muscle strength, logistic regression models were applied. Odds ratios (ORs) and their corresponding 95% confidence intervals (CIs) were calculated in sex-stratified models adjusted for age. The ROC analysis aimed to evaluate the discriminatory ability (AUC) and optimal thresholds of anthropometric measurements for identifying individuals with low muscle strength, while the logistic regression analysis provided a measure of association between these indices and muscle strength status.

Internal validation of the AUC was performed using a bootstrapping procedure, and Harrell’s C-index was calculated from 200 samples drawn with replacement from the original data. Detailed results of this analysis are provided in the Supplementary Materials.

A *p*-value < 0.05 was considered statistically significant in all analyses.

## 3. Results

### 3.1. Characteristics of the Study Population

In the group with low muscle strength, patients were significantly older (82.0 vs. 76.0 years) and had a longer hospital stay (11.00 vs. 10.00 days) compared to those with normal muscle strength. They also had a higher prevalence of stroke (16.4%), neurodegenerative diseases such as Alzheimer’s disease (20.9%) and dementia (26.9%), as well as anemia (40.3%). Additionally, individuals with low muscle strength showed lower total protein levels (62.25 vs. 65.55 g/L) and higher C-reactive protein concentrations (3.5 vs. 2.1 mg/L) (Table 2).

### 3.2. Comparison of Anthropometric and Body Composition Parameters Between Participants with and Without Low Muscle Strength

Analysis of anthropometric and body composition parameters revealed significant differences between participants with normal and low muscle strength. Individuals with low muscle strength exhibited significantly lower body weight (median 64.00 kg vs. 71.70 kg, *p* < 0.001) compared to those with normal muscle strength. Additionally, arm circumference (mean 26.9 vs. 29.38 cm, *p* < 0.001) and calf circumference (median 33.0 vs. 35.0 cm, *p* < 0.001) were significantly smaller in participants with low muscle strength. Body mass index (BMI) and fat mass index (FMI) were also lower in this group (BMI: median 25.7 vs. 28.0 kg/m^2^, *p* = 0.002; FMI: median 7.76 vs. 9.69 kg/m^2^, *p* < 0.001).

Moreover, measures of muscle mass and fat-free mass were significantly decreased among participants with low muscle strength (muscle mass: median 40.5 vs. 43.0 kg, *p* = 0.001; fat-free mass: median 42.7 vs. 45.3 kg, *p* = 0.001). Anthropometric and body composition measurements are presented in Table 3.

### 3.3. Correlations Between Anthropometric Measurements, Anthropometric Indices, Body Composition Parameters, and Handgrip Strength

Spearman correlation analysis (Table 4) showed a significant negative association between age and maximum hand grip strength in the total group (R = −0.44, *p* < 0.001), as well as in women (R = −0.55, *p* < 0.001) and men (R = −0.61, *p* < 0.001). Weight was moderately positively correlated with grip strength (total: R = 0.44, *p* < 0.001). Muscle mass and fat-free mass showed strong positive correlations with grip strength in the total group (R = 0.65 for both, *p* < 0.001), with weaker but significant correlations observed in women and men separately. Notably, body fat percentage correlated negatively with grip strength in the total sample (R = −0.21, *p* < 0.001) but showed a positive correlation in women (R = 0.24, *p* < 0.001). Other anthropometric indices such as BMI, arm circumference, and calf circumference were positively correlated with grip strength, while visceral fat rating showed a moderate positive correlation only in the total group (R = 0.42, *p* < 0.001).

### 3.4. Age-Adjusted Odds Ratios for the Association of Anthropometric Measurements and Indices with Low Muscle Strength in Hospitalized Geriatric Patients, Stratified by Sex

Table 5 presents age-adjusted odds ratios (ORs) for associations between selected anthropometric measurements and indices and low muscle strength in older women and men. Body weight was inversely associated with low muscle strength in both women (OR = 0.964; *p* < 0.001) and men (OR = 0.930; *p* = 0.008). In both sexes, greater arm circumference (women: OR = 0.913; *p* = 0.041; men: OR = 0.793; *p* = 0.007) and greater calf circumference (women: OR = 0.884; *p* = 0.021; men: OR = 0.769; *p* = 0.009) were significantly associated with a lower likelihood of low muscle strength. Fat-free mass and muscle mass also demonstrated inverse associations in women (*p* = 0.007; *p* = 0.008) and men (*p* = 0.019 for both sexes). Traditional indicators of central obesity and fat distribution (BAI, VAI, WHR, WHtR, BRI, body fat percentage, and visceral fat rating) were not significantly associated of low muscle strength (all *p* > 0.05).

### 3.5. Determination of Optimal Cut-Off Values for Identifying Low Muscle Strength Using Receiver Operating Characteristic (ROC) Curve Analysis of Anthropometric Measurements and Indices

**Figure 2:**
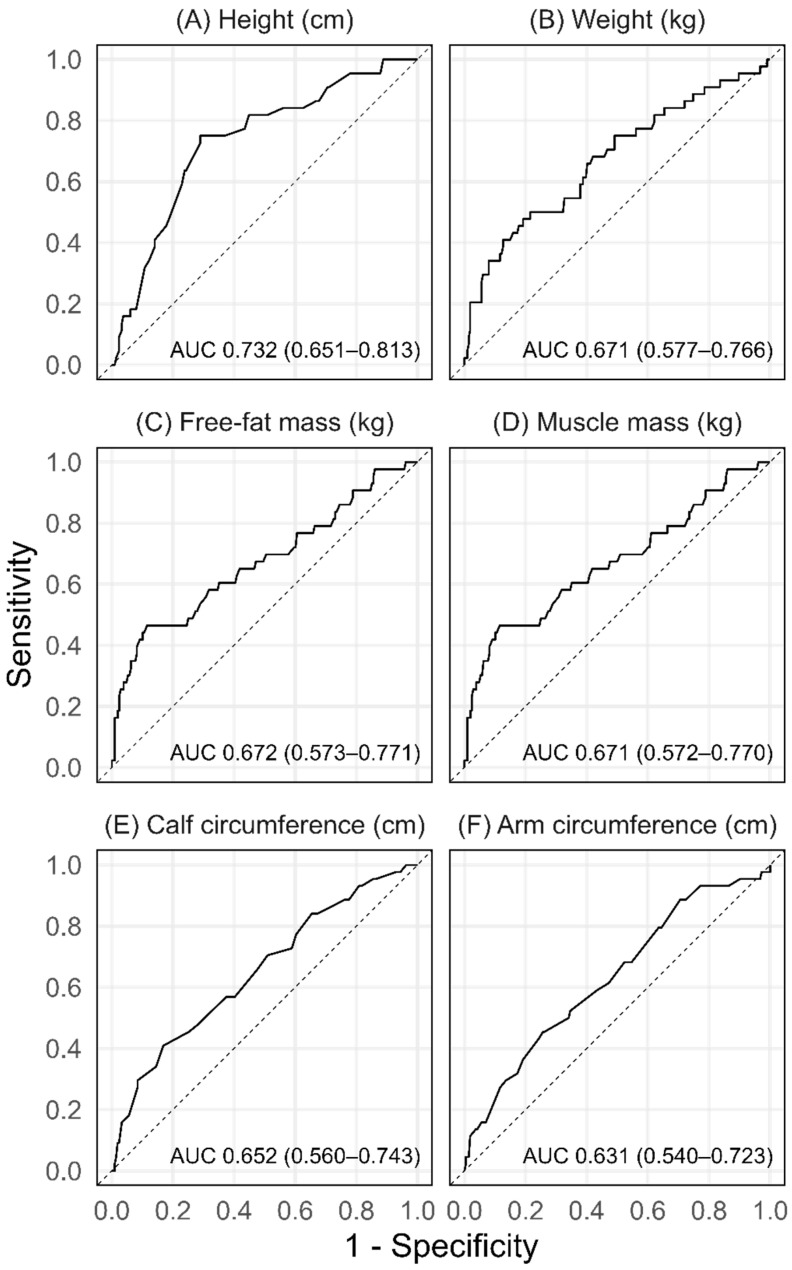
Receiver operating characteristic (ROC) curves of anthropometric measurements and indices for identifying low muscle strength in women for: (**A**) height; (**B**) weight; (**C**) fat-free mass; (**D**) muscle mass; (**E**) calf circumference; (**F**) arm circumference.

In the ROC curve analysis of women (Table 6, Figure 2), height demonstrated the highest discriminative ability (AUC = 0.73; 95% CI: 0.65–0.81), with a sensitivity of 75% and a specificity of 71%, indicating moderate performance in identifying individuals with low muscle strength. Similarly, moderate discriminative ability was observed for body weight (AUC = 0.67), fat-free mass (AUC = 0.67), muscle mass (AUC = 0.67), as well as calf circumference (AUC = 0.65) and arm circumference (AUC = 0.63). These parameters demonstrated relatively high specificity (74–89%) but lower sensitivity (41–48%). Body fat percentage showed the lowest discriminative ability (AUC = 0.40; 95% CI: 0.31–0.50), suggesting limited usefulness for identifying individuals with low muscle strength.

**Figure 3:**
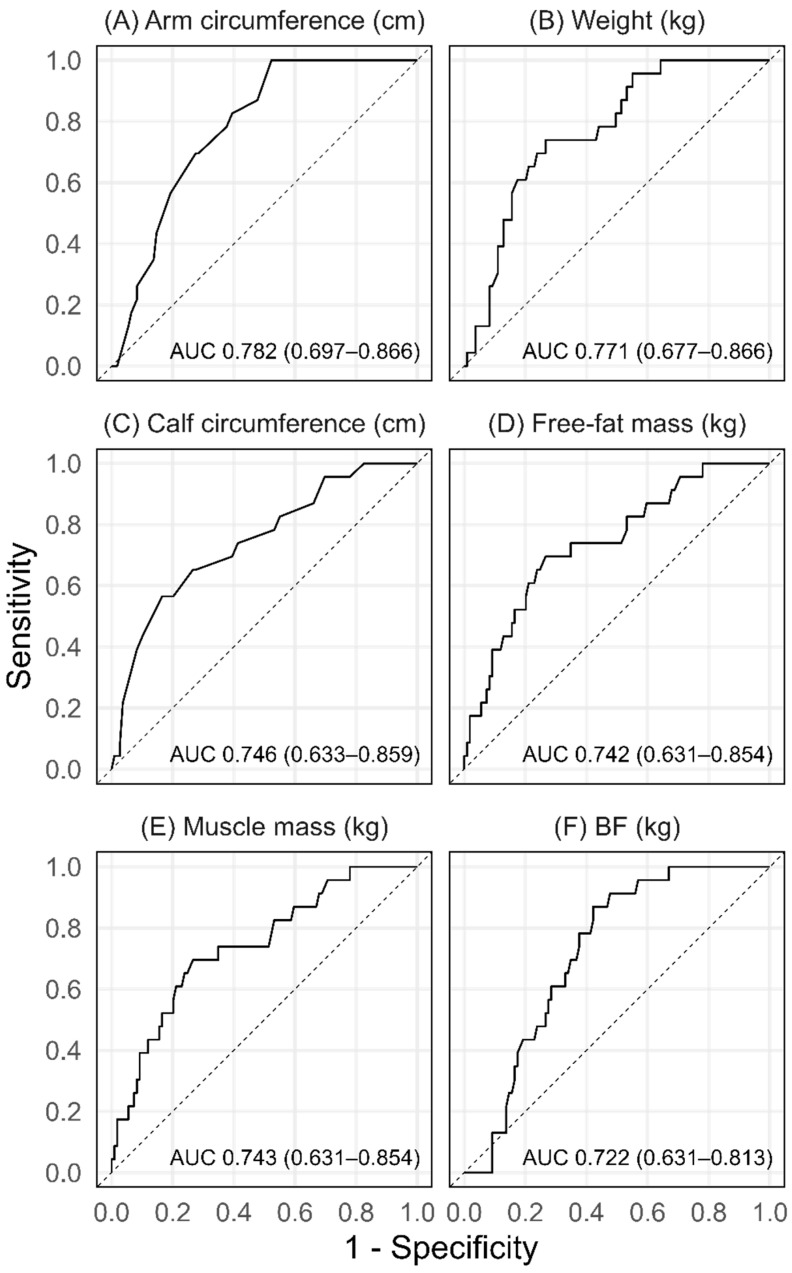
Receiver operating characteristic (ROC) curves of anthropometric measurements and indices for identifying low muscle strength in men for: (**A**) arm circumference; (**B**) weight; (**C**) calf circumference; (**D**) fat-free mass; (**E**) muscle mass; (**F**) body fat.

In the ROC analysis of men (Table 7, Figure 3), arm circumference showed the highest discriminative ability for identifying low muscle strength (AUC = 0.78; 95% CI: 0.69–0.87), with a sensitivity of 100% and a specificity of 48%, suggesting its potential usefulness as a screening measure. Similarly, body weight (AUC = 0.77; 95% CI: 0.68–0.87) and calf circumference (AUC = 0.75; 95% CI: 0.63–0.86) also showed good discriminative ability, with calf circumference demonstrating high specificity (84%) and moderate sensitivity (57%). Fat-free mass and muscle mass also showed moderate discriminative ability (both AUC = 0.74; 95% CI: 0.63–0.85).

Fat mass (kg), fat mass index (FMI), BMI, and waist circumference also showed moderate discriminative ability (AUC = 0.66–0.72), suggesting their potential usefulness for screening low muscle strength.

In contrast, indices such as WHR, WHtR, VAI, BAI, BRI, and visceral fat rating showed low AUC values (0.51–0.57), limiting their clinical usefulness for identifying individuals with low muscle strength.

Detailed ROC-derived thresholds of the anthropometric indices for identifying low muscle strength, including the bootstrapped area under the receiver operating characteristic (ROC) curve (AUC) and Harrell’s C-index for women and men, are provided in the Supplementary Materials (Tables S1 and S2).

## 4. Discussion

The present study evaluated the screening performance of selected anthropometric measurements and indices for identifying EWGSOP2-defined low muscle strength in hospitalized older adults. The main findings indicate that simple limb circumference measurements, particularly arm and calf circumferences, were associated with low muscle strength and showed moderate discriminative ability in ROC curve analysis. In contrast, adiposity-related indices, including WHR, WHtR, BAI, VAI, and visceral fat rating, showed limited usefulness for identifying low muscle strength.

The results of the study indicate an association between age and muscle strength in older adults. The correlations were negative and slightly stronger in men than in women (R = −0.61 vs. R = −0.55), suggesting that handgrip strength decreases with advancing age, which is consistent with previous studies [30] This finding is therefore not novel in itself, but it may have practical relevance, as older age should be considered when interpreting screening results for low muscle strength. ROC curve analysis suggested that age had some discriminatory ability for identifying low muscle strength in this sample, with cut-off values of 76 years for women and 77 years for men. The relatively high sensitivity (86–87%) but modest specificity indicates that age may help identify individuals at higher risk, although it should not be used as a stand-alone screening criterion. Moreover, muscle mass and fat-free mass were most strongly correlated with handgrip strength. However, their routine assessment may be limited in some clinical and care settings because it requires body composition equipment. Although such devices are used in scientific research and increasingly in hospitals, they may not be routinely available in all facilities caring for older adults. Body fat indicators, such as body fat percentage, showed weak or even opposite relationships with muscle strength, suggesting limited usefulness for identifying low muscle strength. Traditional measures of central obesity and fat distribution, such as WHR, WHtR, BAI, or VAI, were not significantly associated with low muscle strength. This may be partly related to sarcopenic obesity, defined as age-related loss of muscle mass and strength or physical performance combined with an increase in fat tissue [31,32,33]. Caregivers and healthcare providers should be aware that obesity, including abdominal obesity, should not be interpreted as protective against low muscle strength. Sarcopenic obesity is associated with serious health risks, including disability, mortality, risk of falls, functional decline, and limited mobility [34].

Analysis of odds ratios (OR) showed that selected anthropometric measurements, such as body weight, BMI, and arm and calf circumferences, were associated with lower odds of low muscle strength. In particular, arm and calf circumferences showed significant inverse associations with low muscle strength, especially in men. In our study, these circumferences were significantly associated with lower odds of low muscle strength in both women (OR arm = 0.913, calf = 0.884) and men (OR arm = 0.793, calf = 0.769; all *p* < 0.01). ROC curve analysis indicated that, in both sexes, simple anthropometric measurements, including arm and calf circumferences, muscle mass, and fat-free mass, showed moderate discriminative ability for identifying low muscle strength (AUC 0.63–0.78). The proposed cut-off values were 26.5 cm for arm circumference and 31.5 cm for calf circumference in women, and 29.5 cm and 33.0 cm, respectively, in men. In our cohort, specificity for calf circumference was 0.83 in women and 0.84 in men, while sensitivity was lower, at 0.41 and 0.57, respectively. These findings indicate that arm and calf circumferences may be useful as simple screening measures, but their moderate AUC values and limited sensitivity, particularly for calf circumference in women, mean that they should not be used to exclude low muscle strength or as stand-alone criteria. Instead, they should be interpreted as preliminary case-finding measures that may help identify patients who require further assessment with handgrip strength testing and, when indicated, full sarcopenia evaluation. These findings are consistent with previous studies suggesting that calf circumference may be useful as a screening marker related to muscle status and low muscle strength, although reported sensitivity and specificity values vary depending on the population, protocol, and cut-off values used [14,15,16,17]. Overall, measuring limb circumferences appears to be a simple and non-invasive approach that may support the preliminary assessment of muscle status in older adults and help identify individuals who require further evaluation, particularly in care settings with limited access to specialized assessment tools.

These findings suggest that simple anthropometric measurements may be useful as an initial screening approach for low muscle strength in older adults. They are easy to perform and do not require specialized equipment for assessing muscle mass or body composition. Their simplicity and time efficiency may make them suitable for initial case-finding in clinical practice and long-term care settings, particularly where access to specialized assessment tools is limited. The results also suggest that measuring these parameters may help identify individuals who require further assessment for sarcopenia according to current clinical guidelines.

In summary, the findings suggest that anthropometric measurements, particularly arm and calf circumferences, may be useful for screening low muscle strength in older adults. Since the risk of low muscle strength increases with age, age should be considered during screening. This approach may facilitate the early identification of older adults who could benefit from further assessment for sarcopenia, particularly in care settings with limited access to specialized equipment and trained personnel. However, the proposed cut-off values should be considered hypothesis-generating rather than diagnostic thresholds for sarcopenia and require external validation in independent populations before routine clinical implementation.

### Study Limitations

Despite the careful selection of the study group, several limitations should be acknowledged. Although the overall sample size was adequate for the main analyses, only a limited proportion of participants were classified as having low muscle strength, which may have reduced the precision of subgroup analyses, particularly in sex-specific comparisons. Increasing the number of participants with low muscle strength in future studies could strengthen the robustness of the findings. Although false discovery rate correction was applied to reduce the risk of type I error due to multiple testing, the exploratory nature of the analyses and the number of statistical comparisons should still be considered when interpreting the findings. Anthropometric measurements were performed only once; therefore, intra-rater reliability could not be assessed, and random measurement error cannot be excluded. The study included hospitalized patients from a single geriatric ward. Although measurements were performed on the first day of hospitalization, the results may not fully reflect the health status and functional capacity of older adults living in the community, receiving primary care, residing in long-term care facilities, or staying in other non-hospital settings. Therefore, the findings and proposed cut-off values should not be directly generalized to these populations without external validation. The authors also acknowledge that cognitive impairment present in some participants may have influenced the results. In the current study, each participant was personally examined by the researcher, who assessed through conversation whether the individual could understand instructions and maintain logical contact sufficient to participate in the study. Participants with cognitive impairment were therefore not automatically excluded; only those whose impairment prevented logical communication and full participation in the study procedures were excluded. The ROC-derived cut-off values were developed and evaluated within the same sample, which may increase the risk of overfitting and limit their generalizability. Due to the limited sample size, split-sample validation was not feasible. Finally, the proposed cut-off values were derived from a single exploratory cohort and should therefore be regarded as hypothesis-generating. They require external validation in independent populations with different clinical characteristics before they can be recommended for routine clinical use.

## 5. Conclusions

The findings of this study suggest that calf and arm circumferences are simple, quick, and non-invasive anthropometric measurements that may be useful for screening low muscle strength in hospitalized geriatric patients, particularly in settings with limited access to specialized equipment. These measurements may help identify individuals who require further assessment for sarcopenia according to current clinical guidelines. Age should also be considered during screening, as the likelihood of low muscle strength increases with advancing age.

The proposed cut-off values should be interpreted as preliminary screening thresholds rather than diagnostic thresholds for sarcopenia. Because they were derived from hospitalized geriatric-ward patients, they require external validation in independent populations, including primary care and long-term care settings, before they can be recommended for routine clinical use.

## Figures and Tables

**Table 1:** Definitions and Formulas of Anthropometric Indices.

Index	Abbreviations	Formulas	Reference
Body Mass Index	BMI	body weight (kg)/height squared (m^2^)	[22]
Body Adiposity Index	BAI	hip circumference (cm)/height (m)^1.5^ − 18	[18]
Waist-to-Hip Ratio	WHR	waist circumference (cm)/hip circumference (cm)	[23]
Waist-to-Height Ratio	WHtR	waist circumference (cm)/height (cm)	[23]
Visceral Adiposity Index	VAI	For women: (waist circumference [cm]/(36.58 + 1.89 × BMI)) × (triglycerides [mmol/L]/0.81) × (1.52/HDL cholesterol [mmol/L])	[19]
		For men: (waist circumference [cm]/(39.68 + 1.88 × BMI)) × (triglycerides [mmol/L]/1.03) × (1.31/HDL cholesterol [mmol/L])	
Fat Mass Index	FMI	body fat (kg)/height squared (m^2^)	[24]
Fat-Free Mass Index	FFMI	fat-free mass (kg)/height squared (m^2^)	[24]
Body Roundness Index	BRI	=365.2 − 365.5 × √(1 − ((waist circumference/2π)^2^/[0.5 × height]^2^))	[25]
Abdominal Volume Index	AVI	[2 × (waist circumference in cm)^2^ + 0.7 × (waist circumference in cm − hip circumference in cm)^2^]/1000	[26]

**Table 2:** Baseline clinical and laboratory characteristics (*N* = 390).

	Normal Muscle Strength (*N* = 323) ^1^	Low Muscle Strength (*N* = 67) ^1^	*p*-Value ^2^	q-Value ^3^
Age (years)	76.0 (70.0–81.0)	82.0 (77.0–85.0)	**<0.001**	**<0.001**
Sex, women *n* (%)	214 (66.3)	44 (65.7)	>0.9	>0.9
Length of hospitalization (days)	10.0 (8.0–13.0)	11.0 (9.0–15.0)	**0.004**	**0.018**
Heart failure *n* (%)	106 (32.8)	23 (34.3)	0.8	>0.9
Coronary artery disease *n* (%)	123 (38.1)	22 (32.8)	0.4	0.7
Prior myocardial infarction *n* (%)	24 (7.4)	5 (7.5)	>0.9	>0.9
Arterial hypertension *n* (%)	272 (84.2)	52 (77.6)	0.2	0.4
Stroke *n* (%)	25 (7.7)	11 (16.4)	0.026	0.091
Alzheimer’s disease *n* (%)	21 (6.5%)	14 (20.9%)	**<0.001**	**0.003**
Dementia *n* (%)	33 (10.2%)	18 (26.9%)	**<0.001**	**0.003**
Depression *n* (%)	20 (6.2%)	4 (6.0%)	>0.9	>0.9
Chronic obstructive pulmonary disease *n* (%)	28 (8.7%)	6 (9.0%)	>0.9	>0.9
Chronic kidney disease (stage 3 or below) *n* (%)	40 (12.4)	13 (19.4)	0.10	0.13
Diabetes mellitus *n* (%)	132 (40.9%)	24 (35.8%)	0.4	0.7
Hypothyroidism *n* (%)	43 (13.3%)	11 (16.4%)	0.5	0.8
Osteoporosis *n* (%)	20 (6.2%)	6 (9.0%)	0.4	0.7
Anemia *n* (%)	78 (24.1%)	27 (40.3%)	**0.007**	**0.027**
Total protein (g/L)	65.55 (61.05–69.00)	62.25 (58.00–64.15)	**<0.001**	**0.004**
Vitamin D (nmol/L)	35.82 (28.20–48.42)	30.45 (24.46–42.68)	0.087	0.2
TSH (uIU/mL)	1.30 (0.78–1.91)	1.16 (0.72–2.21)	0.9	>0.9
Fasting glucose (mmol/L)	5.49 (4.94–6.55)	5.44 (5.05–6.49)	0.7	>0.9
Total cholesterol (mmol/L)	4.61 (3.91–5.54)	4.57 (3.78–5.52)	0.8	>0.9
LDL cholesterol (mmol/L)	2.66 (2.05–3.48)	2.78 (1.97–3.54)	>0.9	>0.9
HDL cholesterol (mmol/L)	1.35 (1.09–1.66)	1.32 (1.09–1.66)	0.6	0.9
Non-HDL cholesterol (mmol/L)	3.24 (2.58–4.00)	3.30 (2.41–4.12)	>0.9	>0.9
Triglycerides (mmol/L)	1.22 (0.93–1.61)	1.14 (0.96–1.44)	0.4	0.7
C-reactive protein (mg/L)	2.10 (0.75–5.00)	3.50 (1.00–22.20)	**0.044**	0.13
eGFR (mL/min/1.73 m^2^)	74.00 (60.00–85.00)	70.00 (53.00–87.00)	0.6	>0.9
Serum creatinine (µmol/L)	75.14 (65.42–89.28)	83.54 (60.11–106.96)	0.12	0.3
Aspartate aminotransferase AST (U/L)	19.00 (16.00–25.00)	17.00 (13.00–20.00)	**<0.001**	**0.004**
Alanine aminotransferase ALT (U/L)	17.00 (13.00–24.00)	15.00 (10.00–19.00)	**<0.001**	**0.004**
Gamma-glutamyl transferase GGTP (U/L)	24.00 (17.00–42.00)	22.50 (15.00–34.00)	0.2	0.4

^1^ Median (Q1, Q3); *n* (%); Mean (SD); ^2^ Pearson’s Chi-squared test; Fisher’s exact test; Wilcoxon rank-sum test; ^3^ False discovery rate correction for multiple testing. TSH, thyroid-stimulating hormone; LDL, low-density lipoprotein; HDL, high-density lipoprotein; AST, aspartate aminotransferase; ALT, alanine aminotransferase; GGTP, gamma-glutamyl transpeptidase.

**Table 3:** Anthropometric and body composition measurement (*N* = 390).

	Normal Muscle Strength (*N* = 323) ^1^	Low Muscle Strength (*N* = 67) ^1^	*p*-Value ^2^	q-Value ^3^
Weight (kg)	71.70 (62.20–81.80)	64.00 (54.00–71.10)	**<0.001**	**<0.001**
Height (cm)	159.00 (153.00–165.00)	151.50 (148.00–160.00)	**<0.001**	**<0.001**
Hip circumference (cm)	101.00 (92.50–110.00)	98.00 (91.00–103.00)	**0.035**	**0.053**
Waist circumference (cm)	104.00 (98.00–112.00)	99.00 (94.00–106.50)	**0.002**	**0.003**
Arm circumference (cm)	29.38 (4.03)	26.90 (3.84)	**<0.001**	**<0.001**
Calf circumference (cm)	35.00 (33.00–38.00)	33.00 (31.00–35.50)	**<0.001**	**<0.001**
Max hand grip strength (kg)	26.50 (22.00–34.00)	16.00 (14.00–24.00)	**<0.001**	**<0.001**
BMI (kg/m^2^)	28.00 (25.30–31.60)	25.70 (23.40–29.20)	**0.002**	**0.003**
BAI (%)	33.87 (29.73–39.15)	34.38 (29.43–40.45)	0.8	0.8
VAI	1.61 (1.13–2.69)	1.66 (1.17–2.30)	>0.9	>0.9
WHR	0.96 (0.07)	0.96 (0.07)	0.6	0.8
WHtR	0.63 (0.58–0.69)	0.63 (0.57–0.68)	0.8	0.8
FFMI index (kg/m^2^)	18.43 (16.80–20.22)	18.25 (16.36–19.49)	0.2	0.2
FMI index (kg/m^2^)	9.69 (7.28–12.64)	7.76 (6.04–10.53)	**<0.001**	**0.002**
BRI	6.24 (5.03–7.56)	6.22 (4.82–7.37)	0.8	0.8
Abdominal volume index (AVI)	20.43 (17.12–24.20)	19.21 (16.58–21.23)	**0.034**	0.053
Body fat (kg)	24.10 (18.50–31.70)	18.45 (13.80–25.20)	**<0.001**	**<0.001**
Body fat (%)	34.80 (28.10–40.40)	30.45 (23.90–38.20)	**0.006**	**0.011**
Muscle mass (kg)	43.00 (38.50–50.80)	40.50 (34.60–46.50)	**0.001**	**0.003**
Fat-free mass (kg)	45.30 (40.60–53.50)	42.70 (36.50–49.00)	**0.001**	**0.003**
Visceral fat rating	12.00 (10.00–16.00)	12.50 (10.00–15.00)	0.6	0.8

^1^ Median (Q1, Q3); Mean (SD); ^2^ Wilcoxon rank-sum test; Welch Two Sample *t*-test; ^3^ False discovery rate correction for multiple testing. BMI, body mass index; BAI, body adiposity index; VAI, visceral adiposity index; WHR, waist-to-hip ratio; WHtR, waist-to-height ratio; FFMI, fat-free mass index; FMI, fat mass index; BRI, body roundness index.

**Table 4:** Correlation between anthropometric parameters and indices and max hand grip strength.

		Total Group			Women Group			Men Group		
		R	*p*-Value	q-Value	R	*p*-Value	q-Value	R	*p*-Value	q-Value
Age	Max hand grip strength (kg)	−0.44	**<0.001**	**<0.001**	−0.55	**<0.001**	**<0.001**	−0.61	**<0.001**	**<0.001**
Weight (kg)		0.44	**<0.001**	**<0.001**	0.31	**<0.001**	**<0.001**	0.44	**<0.001**	**<0.001**
Height (cm)		0.70	**<0.001**	**<0.001**	0.45	**<0.001**	**<0.001**	0.48	**<0.001**	**<0.001**
BMI (kg/m^2^)		0.09	0.071	0.082	0.14	**0.028**	**0.048**	0.28	**0.001**	**0.003**
BAI (%)		−0.43	**<0.001**	**<0.001**	−0.09	0.166	0.232	−0.16	0.073	0.096
VAI		0.03	0.542	0.542	<0.001	0.989	0.989	0.12	0.174	0.215
FFMI index (kg/m^2^)		0.34	**<0.001**	**<0.001**	−0.04	0.494	0.546	0.19	**0.032**	**0.048**
FMI index (kg/m^2^)		−0.10	**0.043**	0.053	0.21	<0.001	**0.001**	0.23	**0.007**	**0.014**
Waist circumference (cm)		0.04	0.414	0.435	0.16	0.009	**0.017**	0.23	**0.007**	**0.014**
Hip circumference (cm)		0.17	**<0.001**	**<0.001**	0.10	0.104	0.156	0.22	**0.010**	**0.017**
Arm circumference (cm)		0.23	**<0.001**	**<0.001**	0.24	**<0.001**	**<0.001**	0.42	**<0.001**	**<0.001**
Calf circumference (cm)		0.32	**<0.001**	**<0.001**	0.23	**<0.001**	**<0.001**	0.51	**<0.001**	**<0.001**
WHR		0.24	**<0.001**	**<0.001**	−0.07	0.292	0.383	0.11	0.208	0.243
WHtR		−0.13	**0.008**	**0.010**	−0.04	0.494	0.546	0.04	0.671	0.671
Body fat (%)		−0.21	**<0.001**	**<0.001**	0.24	**<0.001**	**<0.001**	0.18	**0.034**	**0.048**
Body fat (kg)		0.08	0.142	0.157	0.30	**<0.001**	**<0.001**	0.33	**<0.001**	**<0.001**
Fat-free mass (kg)		0.65	**<0.001**	**<0.001**	0.28	**<0.001**	**<0.001**	0.45	**<0.001**	**<0.001**
Muscle mass (kg)		0.65	**<0.001**	**<0.001**	0.29	**<0.001**	**<0.001**	0.45	**<0.001**	**<0.001**
Visceral fat rating		0.42	**<0.001**	**<0.001**	0.04	0.575	0.604	0.04	0.635	0.671
BRI		−0.13	**0.008**	**0.010**	−0.04	0.494	0.546	0.04	0.671	0.671
AVI		0.17	**<0.001**	**0.001**	0.17	**0.097**	0.156	0.17	**0.010**	**0.017**

R—the Spearman rank correlation coefficient. BMI, body mass index; BAI, body adiposity index; VAI, visceral adiposity index; WHR, waist-to-hip ratio; WHtR, waist-to-height ratio; FFMI, fat-free mass index; FMI, fat mass index; BRI, body roundness index; AVI, abdominal volume index.

**Table 5:** Age-adjusted odds ratios (95% confidence intervals) for the association of anthropometric measurements and indices with low muscle strength in older adults, stratified by sex.

	Women Group			Men Group		
	OR	95% CI	*p*	OR	95% CI	*p*
Weight (kg)	0.964	0.936–0.992	**<0.001**	0.930	0.878–0.978	**0.008**
Height (cm)	0.900	0.844–0.957	**<0.001**	0.939	0.874–1.005	0.072
BMI (kg/m^2^)	0.959	0.894–1.023	0.214	0.883	0.764–1.008	0.076
BAI (%)	1.002	0.951–1.053	0.943	1.013	0.909–1.124	0.805
VAI	1.051	0.833–1.283	0.649	0.944	0.655–1.223	0.705
FFMI index (kg/m^2^)	0.942	0.791–1.104	0.479	0.847	0.632–1.115	0.246
FMI index (kg/m^2^)	0.922	0.840–1.008	0.082	0.835	0.670–1.008	0.081
Waist circumference (cm)	0.974	0.943–1.004	0.103	0.950	0.881–1.018	0.164
Hip circumference (cm)	0.981	0.954–1.007	0.163	0.982	0.937–1.027	0.433
Arm circumference (cm)	0.913	0.835–0.994	**0.041**	0.793	0.662–0.929	**0.007**
Calf circumference (cm)	0.884	0.793–0.978	**0.021**	0.769	0.625–0.930	**0.009**
WHR	0.811	0.006–106.45	0.933	6.097	0.002–22.09	0.662
WHtR	0.431	0.008–20.83	0.675	1.014	0.001–1.083	0.997
Body fat (%)	1.006	0.993–1.023	0.344	0.951	0.881–1.022	0.181
Body fat (kg)	0.956	0.918–0.992	**0.022**	0.918	0.842–0.988	**0.035**
Fat-free mass (kg)	0.891	0.816–0.965	**0.007**	0.896	0.813–0.978	**0.019**
Muscle mass (kg)	0.887	0.809–0.965	**0.008**	0.891	0.805–0.977	**0.019**
Visceral fat rating	0.927	0.815–1.048	0.236	0.865	0.734–0.999	0.063
BRI	0.945	0.831–1.132	0.742	0.985	0.724–1.323	0.920
AVI	0.956	0.892–1.020	0.194	0.947	0.838–1.059	0.352

BMI, body mass index; BAI, body adiposity index; VAI, visceral adiposity index; WHR, waist-to-hip ratio; WHtR, waist-to-height ratio; FFMI, fat-free mass index; FMI, fat mass index; BRI, body roundness index; AVI, abdominal volume index.

**Table 6:** Receiver Operating Characteristic (ROC) analysis and proposed cut-off values of anthropometric measurements and indices for identifying low muscle strength in women.

Parameters	Parameters Cut-Off	AUC (95%CI)	Sensitivity	Specificity	PPV	NPV
Age (years)	76	0.71 (0.63–0.79)	0.86	0.45	0.24	0.94
Weight (kg)	57.1	0.67 (0.58–0.77)	0.48	0.81	0.34	0.88
Height (cm)	151.5	0.73 (0.65–0.81)	0.75	0.71	0.35	0.93
BMI (kg/m^2^)	26.0	0.59 (0.49–0.69)	0.52	0.69	0.26	0.88
BAI (%)	39.64	0.53 (0.43–0.62)	0.46	0.66	0.22	0.86
VAI	1.97	0.48 (0.39–0.57)	0.68	0.38	0.19	0.85
FFMI index (kg/m^2^)	15.73	0.53 (0.43–0.64)	0.28	0.85	0.28	0.85
FMI index (kg/m^2^)	8.08	0.61 (0.52–0.71)	0.44	0.79	0.31	0.87
Waist circumference (cm)	99	0.61 (0.51–0.70)	0.50	0.72	0.27	0.88
Hip circumference (cm)	103	0.56 (0.47–0.65)	0.79	0.37	0.21	0.89
Arm circumference (cm)	26.5	0.63 (0.54–0.72)	0.46	0.74	0.27	0.87
Calf circumference (cm)	31.5	0.65 (0.56–0.74)	0.41	0.83	0.33	0.87
WHR	0.93	0.54 (0.44–0.64)	0.66	0.47	0.20	0.87
WHtR	0.61	0.51 (0.42–0.61)	0.75	0.36	0.19	0.87
Body fat (%)	50.5	0.40 (0.31–0.5)	0.05	0.99	0.50	0.83
Body fat (kg)	29.5	0.65 (0.56–0.74)	0.91	0.36	0.23	0.95
Fat-free mass (kg)	37.1	0.67 (0.57–0.77)	0.47	0.89	0.46	0.89
Muscle mass (kg)	35.2	0.67 (0.57–0.77)	0.47	0.89	0.46	0.89
Visceral fat rating	6	0.53 (0.43–0.63)	0.14	0.98	0.55	0.85
BRI	5.682	0.51 (0.42–0.61)	0.75	0.36	0.19	0.87
AVI	21.3	0.56 (0.47–0.65)	0.77	0.40	0.21	0.90

AUC—area under the curve; CI—confidence interval; PPV—positive predictive value; NPV—negative predictive value; ROC—receiver-operating characteristic. BMI, body mass index; BAI, body adiposity index; VAI, visceral adiposity index; WHR, waist-to-hip ratio; WHtR, waist-to-height ratio; FFMI, fat-free mass index; FMI, fat mass index; BRI, body roundness index; AVI, abdominal volume index.

**Table 7:** Receiver Operating Characteristic (ROC) analysis and proposed cut-off values of anthropometric measurements and indices for identifying low muscle strength in men.

Parameters	Parameters Cut-Off	AUC (95%CI)	Sensitivity	Specificity	PPV	NPV
Age (years)	77	0.73 (0.64–0.83)	0.87	0.56	0.29	0.95
Weight (kg)	71.1	0.77 (0.68–0.87)	0.73	0.73	0.37	0.93
Height (cm)	163	0.69 (0.57–0.83)	0.65	0.75	0.36	0.91
BMI (kg/m^2^)	29.6	0.68 (0.57–0.79)	0.96	0.38	0.24	0.98
BAI (%)	28.17	0.51 (0.38–0.64)	0.74	0.39	0.20	0.88
VAI	2.50	0.54 (0.40–0.67)	0.90	0.29	0.20	0.94
FFMI index (kg/m^2^)	19.09	0.64 (0.50–0.78)	0.57	0.73	0.31	0.89
FMI index (kg/m^2^)	7.98	0.68 (0.58–0.78)	0.87	0.49	0.26	0.95
Waist circumference (cm)	103	0.66 (0.55–0.77)	0.78	0.48	0.24	0.91
Hip circumference (cm)	104.5	0.64 (0.53–0.75)	0.87	0.41	0.24	0.94
Arm circumference (cm)	29.5	0.78 (0.69–0.87)	1.00	0.48	0.29	1.00
Calf circumference (cm)	33	0.75 (0.63–0.86)	0.57	0.84	0.42	0.90
WHR	0.99	0.57 (0.44–0.69)	0.65	0.61	0.26	0.89
WHtR	0.596	0.56 (0.44–0.68)	0.61	0.63	0.26	0.89
Body fat (%)	27.9	0.65 (0.54–0.76)	0.87	0.48	0.26	0.95
Body fat (kg)	19.9	0.72 (0.63–0.97)	0.87	0.58	0.30	0.96
Fat-free mass (kg)	53.1	0.74 (0.63–0.85)	0.69	0.73	0.36	0.92
Muscle mass (kg)	50.4	0.74 (0.63–0.85)	0.69	0.73	0.36	0.92
Visceral fat rating	18	0.62 (0.52–0.72)	0.96	0.31	0.23	0.97
BRI	5.35	0.56 (0.44–0.68)	0.61	0.63	0.26	0.89
AVI	21.9	0.64 (0.5–0.75)	0.87	0.41	0.24	0.94

AUC—area under the curve; CI—confidence interval; PPV—positive predictive value; NPV—negative predictive value; ROC—receiver-operating characteristic. BMI, body mass index; BAI, body adiposity index; VAI, visceral adiposity index; WHR, waist-to-hip ratio; WHtR, waist-to-height ratio; FFMI, fat-free mass index; FMI, fat mass index; BRI, body roundness index; AVI, abdominal volume index.

## Data Availability

The data are available from the corresponding author upon reasonable request.

## Supplementary Materials

The following supporting information can be downloaded at: https://www.mdpi.com/article/10.3390/jcm15145420/s1, Table S1: ROC-Derived Thresholds of Anthropometric Indices for Low Muscle Strength in Women (Bootstrapped AUC and Harrell’s C-index); Table S2: ROC-Derived Thresholds of Anthropometric Indices for Low Muscle Strength in Men (Bootstrapped AUC and Harrell’s C-index).

## Abbreviations

The following abbreviations are used in this manuscript:

EWGSOP	The European Working Group on Sarcopenia in Older People
ICD-10	The International Classification of Diseases
EGFR	Estimated glomerular filtration rate
AST	Aspartate aminotransferase
ALT	Alanine aminotransferase (ALT)
GGT	Gamma-glutamyl transferase
TSH	Thyroid-stimulating hormone
BMI	Body Mass Index
BAI	Body Adiposity Index
WHR	Waist-to-Hip Ratio
WHtR	Waist-to-Height Ratio
VAI	Visceral Adiposity Index
FMI	Fat Mass Index
FFMI	Fat-Free Mass Index
BRI	Body Roundness Index
AVI	Abdominal Volume Index
