# Supplementary material for: Screening Performance of Anthropometric Indices and Determination of Optimal Cut-Off Values for Identifying Low Muscle Strength in Hospitalized Geriatric Patients
Source: J Clin Med. 2026 Jul 10;15(14):5420. doi: 10.3390/jcm15145420 (PMC13412578; doi:10.3390/jcm15145420)
Supplement: Supplementary file 1 [file jcm-15-05420-s001.zip › jcm-4373255-supplementary.pdf]

Supplemental Table S1. ROC-Derived Thresholds of Anthropometric Indices for Low Muscle Strength in Women (Boot-strapped AUC and Harrell's C-index).

Parameters	Parameters cut-off	AUC (95%CI)	Bootstrap validated c-index	Sensitivity (%)	Specificity (%)	PPV (%)	NPV (%)
Age (years)	76	0.71 (0.63-0.79)	0.71 (0.62-0.79)	0.86	0.45	0.24	0.94
Weight (kg)	57.1	0.67 (0.58-0.77)	0.67 (0.58-0.77)	0.48	0.81	0.34	0.88
Height (cm)	151.5	0.73 (0.65-0.81)	0.73 (0.65-0.82)	0.75	0.71	0.35	0.93
BMI (kg/m^2)	26.0	0.59 (0.49-0.69)	0.59 (0.49-0.69)	0.52	0.69	0.26	0.88
BAI (%)	39.64	0.53 (0.43-0.62)	0.52 (0.43-0.57)	0.46	0.66	0.22	0.86
VAI	1.97	0.48 (0.39-0.57)	0.44 (0.35-0.52)	0.68	0.38	0.19	0.85
FFMI index (kg/m2)	15.73	0.53 (0.43-0.64)	0.52 (0.44-0.59)	0.28	0.85	0.28	0.85
FMI index (kg/m2)	8.08	0.61 (0.52-0.71)	0.61 (0.52-0.71)	0.44	0.79	0.31	0.87
Waist circumference (cm)	99	0.61 (0.51-0.70)	0.61 (0.50-0.71)	0.5	0.72	0.27	0.88
Hip circumference (cm)	103	0.56 (0.47-0.65)	0.56 (0.46-0.64)	0.79	0.37	0.21	0.89
Arm circumference (cm)	26.5	0.63 (0.54-0.72)	0.63 (0.53-0.72)	0.46	0.74	0.27	0.87
Calf circumference (cm)	31.5	0.65 (0.56-0.74)	0.65 (0.55-0.73)	0.41	0.83	0.33	0.87
WHR	0.93	0.54 (0.44-0.34)	0.53 (0.44-0.59)	0.66	0.47	0.20	0.87
WHtR	0.61	0.51 (0.42-0.61)	0.49 (0.41-0.54)	0.75	0.36	0.19	0.87
Body fat (%)	50.5	0.40 (0.31-0.5)	0.33 (0.05-0.49)	0.05	0.99	0.5	0.83
Body fat (kg)	29.5	0.65 (0.56-0.74)	0.65 (0.56-0.74)	0.91	0.36	0.23	0.95
Free fat mass (kg)	37.1	0.67 (0.57-0.77)	0.67 (0.58-0.78)	0.47	0.89	0.46	0.89
Muscle mass (kg)	35.2	0.67 (0.57-0.77)	0.67 (0.59-0.76)	0.47	0.89	0.46	0.89
Visceral fat rating	6	0.53 (0.43-0.63)	0.51 (0.43-0.57)	0.14	0.98	0.55	0.85
BRI	5.682	0.51 (0.42-0.61)	0.49 (0.43-0.55)	0.75	0.36	0.19	0.87
AVI	21.3	0.56 (0.47 - 0.65)	0.56 (0.47-0.64)	0.77	0.40	0.21	0.90
	AUC – area under the curve; CI – confidence interval; PPV – positive predictive value; NPV – negative predictive value; ROC – receiver-operating characteristic						

Supplemental Table S2. ROC-Derived Thresholds of Anthropometric Indices for Low Muscle Strength in Men (Bootstrapped AUC and Harrell's C-index)

Parameters	Parameters cut-off	AUC (95%CI)	Bootstrap validated c-index	Sensitivity (%)	Specificity (%)	PPV (%)	NPV (%)
Age (years)	77	0.73 (0.64-0.83)	0.73 (0.65-0.83)	0.87	0.56	0.29	0.95
Weight (kg)	71.1	0.77 (0.68-0.87)	0.77 (0.67-0.88)	0.73	0.73	0.37	0.93
Height (cm)	163	0.69 (0.57-0.83)	0.70 (0.57-0.84)	0.65	0.75	0.36	0.91
BMI (kg/m^2)	29.6	0.68 (0.57-0.79)	0.68 (0.57-0.79)	0.96	0.38	0.24	0.98
BAI (%)	28.17	0.51 (0.38-0.64)	0.46 (0.37-0.53)	0.74	0.39	0.20	0.88
VAI	2.50	0.54 (0.40-0.67)	0.53 (0.40-0.65)	0.90	0.29	0.20	0.94
FFMI index (kg/m2)	19.09	0.64 (0.50-0.78)	0.64 (0.49-0.77)	0.57	0.73	0.31	0.89
FMI index (kg/m2)	7.98	0.68 (0.58-0.78)	0.68 (0.58-0.77)	0.87	0.49	0.26	0.95
Waist circumference (cm)	103	0.66 (0.55-0.77)	0.67 (0.55-0.78)	0.78	0.48	0.24	0.91
Hip circumference (cm)	104.5	0.64 (0.53-0.75)	0.64 (0.53-0.76)	0.87	0.41	0.24	0.94
Arm circumference (cm)	29.5	0.78 (0.69-0.87)	0.78 (0.69-0.88)	1	0.48	0.29	1
Calf circumference (cm)	33	0.75 (0.63-0.86)	0.74 (0.63-0.86)	0.57	0.84	0.42	0.90
WHR	0.99	0.57 (0.44-0.69)	0.57 (0.45-0.70)	0.65	0.61	0.26	0.89
WHtR	0.596	0.56 (0.44-0.68)	0.54 (0.43-0.65)	0.61	0.63	0.26	0.89
Body fat (%)	27.9	0.65 (0.54-0.76)	0.65 (0.55-0.76)	0.87	0.48	0.26	0.95
Body fat (kg)	19.9	0.72 (0.63-0.97)	0.72 (0.62-0.81)	0.87	0.58	0.30	0.96
Free fat mass (kg)	53.1	0.74 (0.63-0.85)	0.75 (0.64-0.88)	0.69	0.73	0.36	0.92
Muscle mass (kg)	50.4	0.74 (0.63-0.85)	0.74 (0.64-0.87)	0.69	0.73	0.36	0.92
Visceral fat rating	18	0.62 (0.52-0.72)	0.62 (0.52-0.71)	0.96	0.31	0.23	0.97
BRI	5.35	0.56 (0.44-0.68)	0.55 (0.43-0.65)	0.61	0.63	0.26	0.89
AVI	21.9	0.64 (0.53 - 0.75)	0.64 (0.53-0.74)	0.87	0.41	0.24	0.94
	AUC – area under the curve; CI – confidence interval; PPV – positive predictive value; NPV – negative predictive value; ROC – receiver-operating characteristic						
